# Prediction of Mechanical Properties of Injection-Molded Weld Lines of Glass Fiber-Reinforced Composites

**DOI:** 10.3390/polym17233120

**Published:** 2025-11-24

**Authors:** Zuguo Bao, Yunxiang Yan, You Zhang, Ruihan Dong, Weijian Han, Qing Liu

**Affiliations:** 1 Key Laboratory for Light-Weight Materials, Nanjing Tech University, Nanjing 210009, China; 2 Yangtze Delta Region Institute of Advanced Materials, Suzhou 215133, China; 3 School of Mechatronics and Vehicle Engineering, Chongqing Jiaotong University, Chongqing 400074, China; 4 School of Mathematics and Physics, Xi’an Jiaotong-Liverpool University, Suzhou 215123, China

**Keywords:** weld lines, glass fiber-reinforced composites, mechanical properties prediction

## Abstract

The weld line has a great impact on the mechanical properties of injection-molded parts, especially large ones. Currently, there is still a lack of useful simulation tools to accurately predict the mechanical properties of weld lines. To solve this issue, this paper studies the mechanical properties of weld lines in injection-molded glass fiber (GF)-reinforced composites and builds a mathematical model to predict these properties. This model combines polymer chain dynamics with fiber–matrix interfacial debonding mechanics, enabling multiscale characterization of weld line strength. The experimental results showed that injection temperature, injection pressure, and fiber content all affect the mechanical properties of weld lines, with fiber content exerting the most significant influence. To predict weld line strength, a mathematical model was established by integrating multiple simulation software and tools: Moldex3D for mold flow analysis, Digimat for material modeling, and Abaqus for multiscale mechanical analysis. Comparisons between the simulation and experimental results demonstrated high accuracy of the model (errors less than 10% for tensile strength and 3.5% for stiffness, respectively), which provides an effective tool for predicting the weld line performance of glass fiber-reinforced polypropylene composites.

## 1. Introduction

Polymers and composites are used increasingly widely in various industries due to their high specific strength, durability, and resistance to extreme conditions, making them ideal for aviation manufacturing, marine engineering, new-energy vehicles, construction, and sports equipment [1,2]. Injection molding remains the preferred manufacturing method for these materials, valued for its high efficiency, precision in complex geometries, and cost-effectiveness [3]. However, the weld line, caused by the convergence of molten polymer flows in the mold, persists as a critical defect, disrupting fiber orientation, weakening fiber–matrix bonds, and impairing the mechanical performance in reinforced composites [4,5]. Understanding weld line mechanics and developing reliable predictive tools are essential for material and component design [6].

Previous studies have shown that higher injection/mold temperatures and pressures enhance weld line strength by improving melt interdiffusion [7,8,9,10,11,12]. Fiber content has complex effects, indicating that moderate levels (10–30 wt% in PP-GF) boost strength, while excess (≥35 wt%) causes agglomeration [13,14,15,16]. Wang et al. [17] found glass fiber-reinforced nylon 66 weld line strength unaffected by fiber content, while Zhong et al. [18] used CAE and cases to show that higher content exacerbates degradation. Livingston et al. [19] used in situ DIC to quantify fiber–matrix interface behavior, revealing interfacial debonding. Fisa et al. [20] found glass fiber (GF)-reinforced PP weld lines form 2–8 mm zones with fibers parallel to the weld plane, and strength depends solely on fiber content. Tran et al. [21] used ANNs to predict PA6/30%GF weld line strength, noting packing pressure and melt temperature effects. Paturi [22] developed an Artificial neural network model (ANN) to estimate the permissible range of the weld line and the sink mark of plastic parts based on the published data from studies.

In addition, there is also some research progress in simulation models. Polat [23] integrated the real-time data on fiber orientation and weld line effects into finite element analysis (FEA) models, leading to better predictions in the performance of the parts. Guo et al. [24] modified a melt diffusion Flory–Huggins model to predict weld line strength of amorphous polymers and blends. Harnnarongchai et al. [25] proposed a modified rule of mixtures to study fiber length/orientation impact on short glass fiber-reinforced PP weld line strength. Ruan et al. [26] developed the first fully coupled multiscale model to explore short-fiber composite cooling crystallization. Pieressa et al. [27] linked PINN output to weld line visibility via FLR, with pre-training reducing experimental demand.

However, existing fiber orientation models fail to address crystallization kinetics–fiber orientation coupling [28]. Current models cannot capture dynamic fiber–matrix interactions [26], and Ullah et al. [29] noted that welding force/amplitude regulates GF/PP interfacial crystallinity, which is poorly characterized. Additionally, injection parameter optimization [30,31,32] lacks integration with multiscale simulation, and the Mori–Tanaka model [33] is not fully incorporated into strength prediction. These gaps demand an integrated approach.

In this study, the mechanical properties and their influencing factors of the weld lines in glass fiber-reinforced polypropylene composites are investigated. Glass fiber-reinforced polypropylene was selected as the subject material due to its wide applications in injection-molded products for many fields. A mathematical model is developed to predict the mechanical properties of weld lines, combining polymer chain dynamics with fiber–matrix interfacial debonding mechanics, which, for the first time, realizes the coupling of semi-crystalline polymer dynamics and long-fiber anisotropy and solves the defects of traditional models that ignore intermolecular chain interactions and crystallization kinetics–fiber orientation coupling. A multiscale simulation framework, which integrates Moldex3D for derived fiber orientation data, Digimat for microstructure reconstruction, and Abaqus for multiscale analysis, clarifies the influence of the laws of injection temperature, pressure, and fiber content on the mechanical properties of weld lines. The experimental and simulation results are compared to validate the accuracy and effectiveness of the developed model and simulation framework.

## 2. Methodology

### 2.1. Materials

Neat polypropylene (PP, SK R370Y) and 30 wt% glass fiber-reinforced polypropylene (PP-30GF, SK G310) were purchased from SK Global Chemical Co. Ltd., Seoul, Republic of Korea. The glass fibers (GFs) added in PP-30GF have a length of 3 mm and a diameter of 20 μm. The basic mechanical properties of the materials are listed in Table 1. Composite pellets with lower fiber contents were prepared by mixing PP-30GF and neat PP in predefined proportions (e.g., PP-20GF and PP-10GF).

### 2.2. Specimen Preparation and Characterizations

Specimens were prepared using an injection molding machine (HAITIAN-MA 900Ⅲ/280-A model), with a maximum clamping force of 90 tons. The injection molding experiments used a controlled-variable method to study the independent effects of injection temperature and pressure. For the temperature tests, the injection temperature was set at 190, 200, 210, 220, and 230 °C, with a fixed intermediate injection pressure of 7.5 MPa (to avoid interference from extreme pressures). For the pressure tests, the injection pressure was adjusted to 2.5, 5, 7.5, 10, and 12.5 MPa, using a constant intermediate injection temperature of 210 °C (to maintain stable melt fluidity). The injection speed was fixed at 60 mm/s for all samples. Prior to processing, all composite pellets were pre-dried at 80 °C for 4 h to remove residual moisture. These dried pellets were subsequently used to fabricate the experimental specimens.

**Figure 1:**
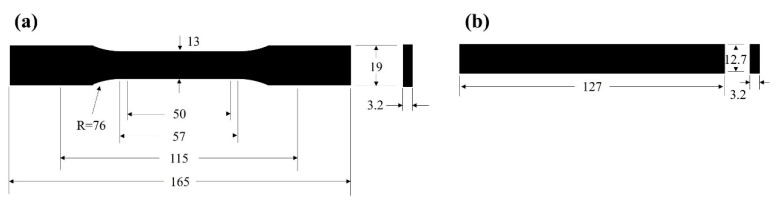
Injection molding specimens: (**a**) tensile testing specimen; (**b**) flexural testing specimen.

As shown in Figure 1a, the tensile specimens were designed according to the ASTM D638 Type I standard. Each specimen had a total length of 165 mm, a gauge length of 50 mm, a width of 13 mm, and a thickness of 3.2 mm. Tensile tests were conducted at a crosshead speed of 5 mm/min. The flexural specimens were designed following the ASTM D790 standard, with a length of 127 mm, a width of 12.7 mm, and a thickness of 3.2 mm. Flexural tests were performed under three-point bending conditions with a span width of 51.2 mm at a loading rate of 1.4 mm/min. Both the tensile and flexural tests were conducted using an INSTRON 5982 testing machine. For all the tests, five duplicate specimens were tested for each material, and the mean values and standard deviations were provided.

**Figure 2:**
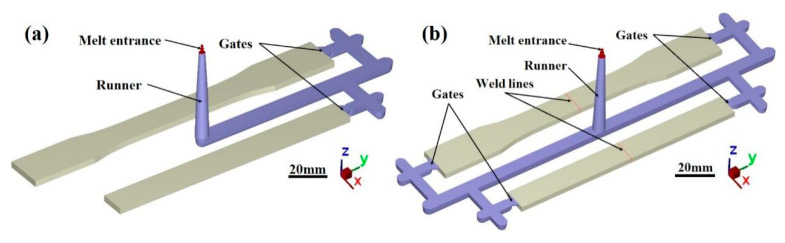
Injection runners of the specimens: (**a**) without weld lines; (**b**) with weld lines.

The injection runner designs of the specimens are shown in Figure 2. The runner design for the specimens without weld lines is shown in Figure 2a, with the gate positioned at one end of the mold cavity. The cold-runner design for the specimens with weld lines is shown in Figure 2b. This configuration allowed the melt to be injected simultaneously from both ends of the mold cavity for tensile and flexural strength tests, creating an intersection region where weld line defects were formed. The transition between the two cold-runner designs was controlled using a valve.

After mechanical testing, the fracture surfaces of specimens were examined using scanning electron microscopy (SEM, ZEISS-EVO25) to further investigate the effects of the weld lines on their mechanical properties.

### 2.3. Simulation Framework

A multiscale simulation framework was developed to predict the mechanical behavior of weld lines in glass fiber-reinforced polypropylene (PP-GF) composites by integrating injection molding process analysis, microstructure reconstruction, and mechano-property prediction. The workflow commenced with simulating the injection molding process using Moldex3D software (version: 2020) to model the filling, packing, and cooling stages. Key parameters critical to weld line formation, including fiber orientation distribution, melt temperature profiles at flow fronts, and weld line formation time, were extracted for subsequent mechanical analysis.

Injection molding is inherently a non-isothermal process due to the temperature and shear rate-dependent viscosity of the polymer melt. The Cross-WLF model [34,35,36,37] described the non-Newtonian behavior of polypropylene under these complex conditions as follows:

(1) $$\eta (\dot{\gamma },T)=\frac{\eta_{0}(T)}{{1+(\eta_{0}\dot{\gamma }/\tau^{*})}^{1-n}}$$

where *η(*$\dot{\gamma }$*,T)* refers to the viscosity of the melt (Pa·s), which changes with the shear rate and temperature. *η*_0_ *(T)* is the zero-shear viscosity as a function of temperature (Pa·s). $\dot{\gamma }$ is the shear rate (s^−1^). $\tau^{*}$ is the critical shear stress (Pa) representing the transition between Newtonian and non-Newtonian flow, and n is the dimensionless power-law index characterizing the degree of shear thinning. This equation captures the dual dependence of viscosity on shear rate and temperature, critical for predicting melt flow behavior and fiber orientation during filling.

To address the limitations of conventional models that decouple polymer chain dynamics from fiber–matrix interactions, the framework integrates two key mechanisms.

1. Polymer chain diffusion was based on the Flory–Huggins free energy theory [37], which governs molecular chain interdiffusion at weld lines as follows:

(2) $$\frac{\sigma_{\omega }}{\sigma_{b}}=1-exp\left[-\frac{C_{0}D}{kT}\left(kT\frac{\rho }{M_{w}}\delta_{x}\cdot F\cdot ln2+2\Gamma_{0}{\left(1-\frac{T}{T_{cr}}\right)}^{\frac{11}{9}}\right)t\right]$$

where *σ_ω_* is the weld line strength, *σ_b_* is the bulk material strength (without weld lines), *δ_x_* is the diffusion depth of polymer chains, T is the absolute temperature, *T_cr_* is the critical temperature, *C*_0_ is a material constant related to chain mobility, *D* is the diffusion coefficient, *k* is the Boltzmann constant, *ρ* is the polymer density, *M_w_* is the weight-average molecular weight, *F* is a geometric factor (material constant), *Γ*_0_ is a fitting parameter (material constant), and *t* is time. This equation quantifies the temperature- and time-dependent healing of weld lines through molecular chain entanglement.

2. Fiber–matrix interfacial debonding, which predicts strength reduction due to fiber alignment parallel to the weld plane [38,39], is given as follows:

(3) $$\sigma_{\omega }=\sigma_{m}\left(1-A_{f}\right)$$

(4) $$A_{f}={\left(4\emptyset /\pi \right)}^{1/2}$$

where *A_f_* is the area fraction occupied by fibers, ∅ is the fiber volume fraction, *σ_ω_* is the weld line strength, and *σ_m_* is the matrix strength (without fiber reinforcement).

The interface diffusion thickness (*t_h_*) [35], a critical parameter linking molecular-scale diffusion to macroscale performance, is defined as follows:

(5) $$t_{h}=\delta_{x}^{2}/2D$$

where *t_h_* is the interface diffusion thickness, *δ_x_* is the diffusion depth of polymer chains, and *D* is the diffusion coefficient. This relationship determines the time required for polymer chains to diffuse across the weld interface, directly influencing interfacial fusion quality.

The weld line factor (*F_kl_*) [36] quantifies mechanical degradation caused by weld lines as follows:

(6) $$F_{kl}=\frac{\sigma_{WL}}{\sigma_{NWL}}$$

where *F_kl_* is the weld line factor, *σ_WL_* is the strength of specimens with weld lines, and *σ_NWL_* is the strength of specimens without weld lines. A smaller *F_kl_* value indicates more severe performance loss, providing a metric for process optimization [17].

The stiffness tensor C of the composite was derived from the volume-averaged stress–strain relationship as follows:

(7) σ=C∶ϵ

where σ and ϵ represent the volume-averaged stress and strain tensors, respectively. To account for the influence of fiber orientation anisotropy, the Mori–Tanaka [33] mean-field homogenization model was employed. This model calculated the orientation-dependent elastic modulus by incorporating the strain concentration tensor A, which depends on fiber aspect ratio and orientation as follows:

(8) $$C_{eff}=C_{m}+f_{f}\left[\left(C_{f}-C_{m}\right)∶A\right]{∶\left[\left(1-f_{f}\right)I+f_{f}A\right]}^{-1}$$

where $C_{m}$ and $C_{f}$ denote the stiffness tensors of the matrix and fiber, respectively, and $f_{f}$ is the fiber volume fraction.

To implement this framework, Moldex3D was employed to simulate fiber distribution, orientation, and thermomechanical fields (e.g., pressure, temperature). These simulation results were then used to guide the generation of representative volume elements (RVEs) via Digimat-FE, which integrated fiber volume fractions (0–30 wt%), fiber aspect ratios (L/D = 200), calibrated interfacial properties fiber volume fraction (0–30 wt%), aspect ratio (L/D = 200), and calibrated interfacial properties. A custom FORTRAN subroutine was embedded in Abaqus coupled polymer chain dynamics (e.g., entanglement, stress relaxation) with fiber–matrix debonding mechanics while enforcing periodic boundary conditions to ensure strain compatibility. During stress analysis, the RVE model was subjected to mechanical loads, generating performance curves that correlate weld line strength with molding conditions.

The seamless integration of Moldex3D (process simulation), Digimat (microstructure reconstruction), and Abaqus (mechano-property prediction) enabled a holistic multiscale analysis. This approach effectively overcame the limitations of conventional models, which usually only consider fiber–fiber interactions yet overlook intermolecular chain interactions in the polymer matrix. In contrast, the model in this work not only incorporates fiber–fiber interactions but also explicitly accounts for intermolecular chain interactions in the matrix based on the explicit inclusion of semi-crystalline polymer dynamics and long-fiber anisotropy.

### 2.4. Modeling

The RVE model in this study adopted C3D4 tetrahedral elements for mesh partitioning, which was suitable for stress analysis of complex structures and had good mesh adaptability and computational stability. During the mesh generation process, the element size was set to 12, and the actual average size of the generated element was 4.6. The entire simulation model consisted of approximately 150,000 elements and 220,000 nodes, and the mesh quality meets the requirements of simulation analysis. Reasonable selection of element type and mesh density configuration ensured accurate reflection of mechanical properties, such as stress distribution in the weld line zone and the interaction between fibers and matrix interface, providing accurate simulation results.

The number of elements and nodes mentioned above was optimized through the investigation of mesh convergence, which was mainly adjusted by the size of the element. In order to study the mesh convergence, three mesh scenarios of different sizes were designed. Scenario 1 generated 650,000 elements and 410,000 nodes. It took a long time to conduct simulations, and we frequently encountered a non-convergence phenomenon after repeated simulations, resulting in errors. Scenario 2 generated 80,000 elements and 120,000 nodes, and its simulation showed a significant increase in speed, but the results deviated significantly from the experimental tests. Scenario 3 used 150,000 units and 220,000 nodes, and its simulation results were stable with relatively small errors. Taking into account both computational accuracy and efficiency, Scenario 3 (150,000 elements, 220,000 nodes) was ultimately selected as the optimal mesh configuration. This configuration ensured the reliability of the simulation results and avoided the significant increase in computational costs caused by excessive mesh refinement.

**Figure 3:**
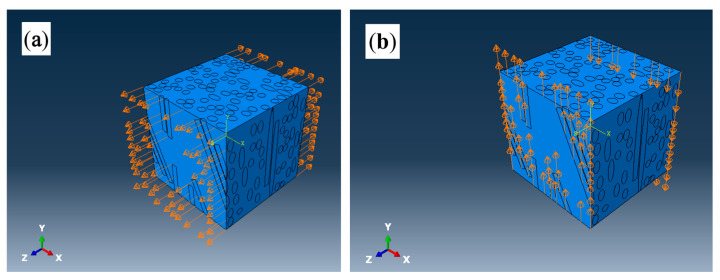
Loading of tensile stress (**a**) and flexural stress (**b**) on the RVE models.

The loading of tensile stress on the RVE model is shown in Figure 3a. The displacements of the front and back planes (perpendicular to the Z-axis) of the RVE model in the U3 direction were set as 100 and −100, respectively. No constraints were applied in other directions. Similarly, the loading of flexural stress on the RVE model is shown in Figure 3b. The displacements of the front and back planes (perpendicular to the Z-axis) of the RVE model in the U2 direction were set as 100 and −100, respectively. And, no constraints were applied in other directions.

**Figure 4:**
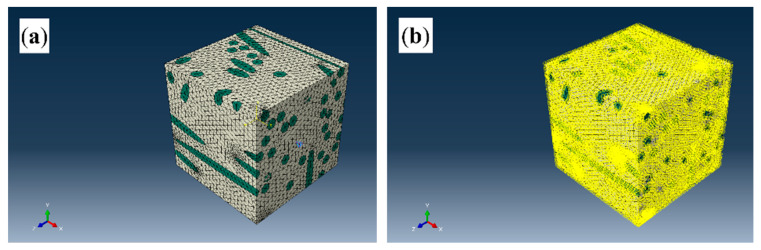
RVE models: (**a**) without periodic boundary conditions; (**b**) with periodic boundary conditions.

To ensure that the RVE model accurately reflects the macroscopic mechanical properties of the specimen, periodic boundary conditions were applied for constraint. Figure 4a shows the RVE model without periodic boundary conditions, in which the green and gray elements represent the glass fiber and PP matrix respectively. And Figure 4b shows the RVE model with periodic boundary conditions. The periodic boundary conditions can effectively eliminate the artificial effects of model boundaries, ensuring consistency between the micro-scale RVE behavior and the macro-composite material properties and improving the reliability of subsequent mechanical simulations.

## 3. Experimental Results and Discussion

### 3.1. Influence of Injection Temperature on Mechanical Properties of Weld Lines

**Figure 5:**
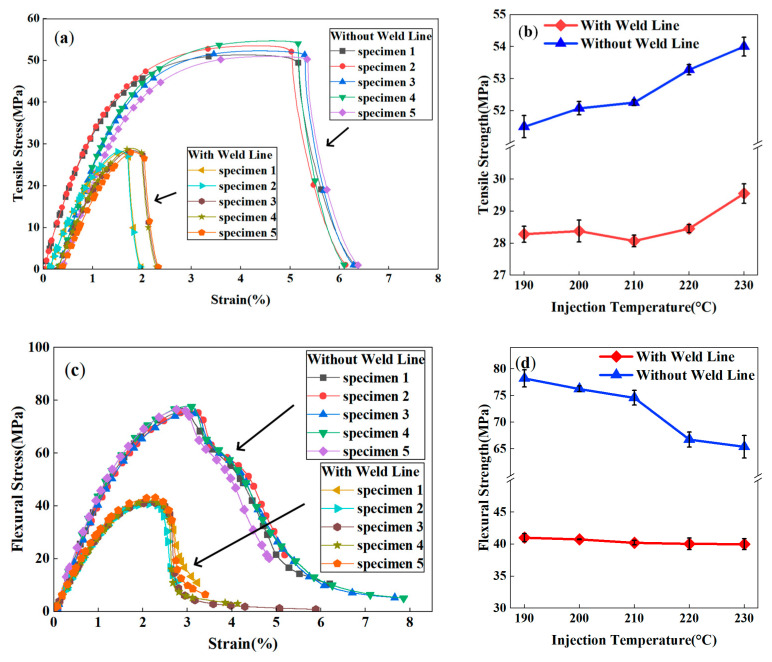
Mechanical properties of PP-20GF composite specimens with or without weld lines: (**a**) tensile stress–strain curves; (**b**) flexural stress–strain curves; (**c**) tensile strength vs. injection temperature; (**d**) flexural strength vs. injection temperature.

Figure 5 illustrates the tensile and flexural properties of PP-20GF composites with and without weld lines as a function of injection temperature (injection pressure 7.5MPa). It can be observed that the existence of the weld lines apparently influenced the mechanical properties of the composites. At 210 °C, the tensile strength of specimens with weld lines was 46.3% lower (28.1 MPa vs. 52.3 MPa) than that of specimens without weld lines, and the flexural strength of the former was only 53.9% of the latter (40.2 MPa vs. 74.6 MPa). This significant decline in mechanical properties induced by weld line defects is very steadily demonstrated by the stress–strain curves of the duplicated specimens in Figure 5a.

For different injection temperatures (Figure 5b), weld line specimens maintained a low strength range of 27.3–29.6 MPa across 190–230 °C, a 45.3–47.4% decrease from specimens without weld lines (ranging from 51.8 to 54.0 MPa). Notably, as the temperature increased from 190 °C to 230 °C, the tensile strength of specimens with weld lines only slightly increased (approximately 2.8%), while specimens without weld lines showed steady strength improvement. The substantial performance gap between the two group specimens remained evident throughout the temperature range.

Similarly, the flexural properties of the composites were also influenced by the weld lines (Figure 5c), demonstrating an obvious declining trend when compared with specimens without weld lines. However, for the injection temperature effect, flexural strength (Figure 5d) showed distinct trends, in which weld line specimens stayed stable at 40.0–41.0 MPa, while specimens without weld lines displayed a clear decreasing trend: dropping from 78.2 MPa at 190 °C to 65.4 MPa at 230 °C. Weld line specimens’ flexural strength was 39.0–48.8% lower than those without weld lines. This temperature-driven trend divergence narrowed the performance gap between specimens with and without weld lines.

**Figure 6:**
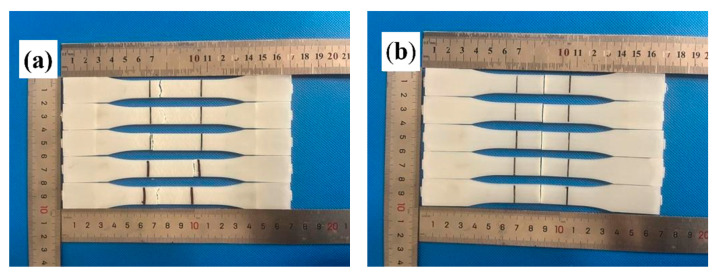

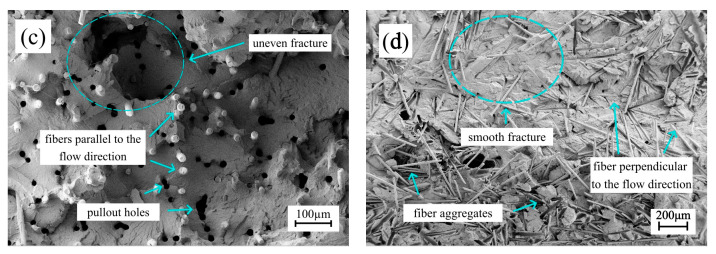
Fracture morphology of PP-20GF tensile specimens: (**a**) macroscopic fracture, without weld lines; (**b**) macroscopic fracture, with weld lines; (**c**) SEM fracture, without weld lines; (**d**) SEM fracture, with weld lines.

Figure 6 compares the fracture surfaces of specimens with and without weld lines. For the specimens without weld lines, the tensile fracture occurred at a random position in the gauge length, as shown in Figure 6a. For the weld line specimens in Figure 6b, neat fracture surfaces occurred precisely at the weld line regions, indicating that the weld line defects were prone to fracture under stress loads. Furthermore, the microscopic fracture morphology of the specimens was also observed by SEM. The specimen without a weld line (Figure 6c) exhibited an uneven fracture surface with fibers aligned parallel to the flow direction, leaving fiber pullout holes, which is evidence of robust load-bearing capacity. In weld line regions (Figure 6d), fibers are aligned perpendicular to the flow direction, forming a smooth “2D” fracture surface with visible fiber grooves. This orientation prevents effective load transfer, as most fibers lie parallel to the fracture plane.

The weld line factor (*F_kl_*) of PP-20GF composites across injection temperatures was calculated in Table 2 to further analyze the temperature’s effect on the mechanical properties of weld lines. *F_kl_* of tensile strength fluctuated between 0.53 and 0.55, showing no visible trend with increasing temperature and indicating that simply raising the injection temperature had little improvement for the tensile strength of weld lines. By contrast, *F_kl_* of flexural strength exhibited a clear positive temperature effect, increasing apparently from 0.52 at 190 °C to 0.61 at 230 °C. This indicates that increasing the injection molding temperature to some extent offsets the adverse effects of decreased weld line properties.

The observed paradoxical thermal responses—simultaneous tensile strength enhancement and flexural performance degradation—highlight unique challenges in semi-crystalline composite processing. Although elevated temperatures improve interfacial fusion through enhanced polymer chain mobility (yielding superior tensile properties) [7], they concurrently trigger matrix embrittlement via accelerated crystallization [26]. This dual-phase interaction, previously undocumented in amorphous polymers or short-fiber composites, has been validated through the multiscale model’s capacity to resolve coupled thermal–mechanical–fiber orientation interactions. Such computational capability enables precise design of PP-GF components with optimized weld line defect tolerance for high-stress applications [25].

### 3.2. Influence of Injection Pressure on the Mechanical Properties of Weld Lines

**Figure 7:**
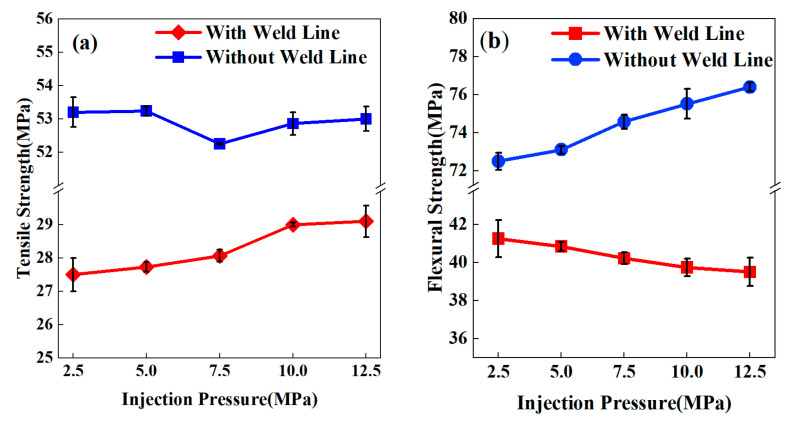
Mechanical properties of PP-GF composite specimens with or without weld lines under different injection pressures: (**a**) tensile strength; (**b**) flexural strength.

Figure 7 illustrates the tensile and flexural strengths of PP-20GF composites under varying injection pressures. For tensile strength (Figure 7a), increasing pressure from 2.5 to 12.5 MPa brought about a steady improvement in weld line specimens, with their tensile strength rising from 27.5 MPa to 29.1 MPa. In contrast, specimens without weld lines maintained stable performance around 53.0 MPa, with a fluctuation range of less than 1.5%.

Conversely, flexural strength (Figure 7b) showed contrasting behaviors between the two specimen types. As pressure increased from 2.5 to 12.5 MPa, specimens without weld lines achieved a significant strength enhancement, climbing from 72.5 MPa to 76.4 MPa (a 5.4% increase). However, weld line specimens experienced a continuous strength decline, dropping from 41.3 MPa to 39.5 MPa (a 4.2% decrease). Throughout the 2.5–12.5 MPa experimental range, the weakening effect of injection pressure on the mechanical properties of weld lines remained obvious.

Table 3 lists the weld line factor (*F_kl_*) of PP-20GF composites under different pressures, to further analyze the pressure’s effect on the mechanical properties of weld lines. *F_kl_* of tensile strength showed a improving trend with increasing injection pressure, rising from 0.52 to 0.55. By contrast, *F_kl_* of flexural strength exhibited a continuous decreasing trend, dropping from 0.57 to 0.52. This reveals that the effect of injection pressure on weld line performance apparently varies across different mechanical properties.

The enhanced tensile performance is attributed to the microstructural evolution, where high-pressure processing promotes fiber interlocking that reinforces the weld zone [17]. However, the accelerated melt-filling rate under elevated pressure inhibits polymer crystallization, progressively diminishing the matrix’s capacity to withstand flexural loading.

The results indicate the critical need for pressure parameter optimization specific to semi-crystalline PP-GF composites. Although increased injection pressures improve tensile strength through enhanced fiber entanglement, they simultaneously amplify brittleness under flexural conditions. To reconcile these competing mechanisms, the multiscale model’s predictive power must be strategically deployed to coordinate fiber dispersion quality with interfacial adhesion strength, ultimately securing reliable weld line functionality in high-pressure operational environments.

### 3.3. Effect of Fiber Content on Mechanical Properties of Weld Lines

**Figure 8:**
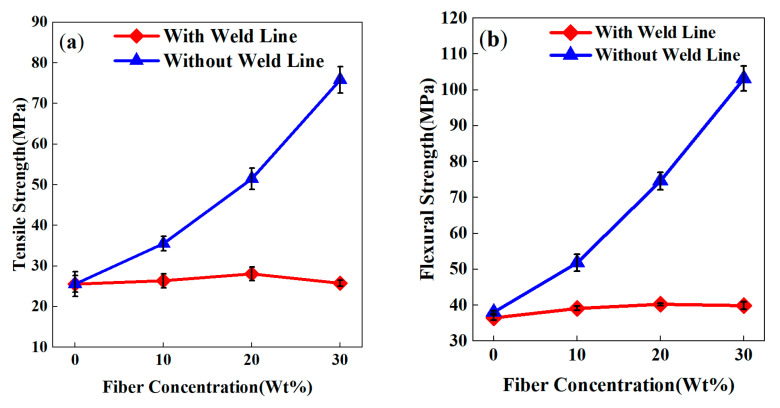
Combined simulation curves of PP-GF composites with weld lines under different fiber contents: (**a**) tensile strength; (**b**) flexural strength.

A pronounced enhancement in tensile and flexural strength of PP-GF composites was observed with increasing GF content, as demonstrated in Figure 8. Fiber content was identified as the dominant factor governing mechanical performance, surpassing the effects of injection temperature and pressure variations. In specimens without weld lines, tensile and flexural strengths exhibited a progressive enhancement with elevated GF content (Figure 8a,b), attributable to microstructural evolution at 210 °C processing conditions. The PP matrix was reinforced through the establishment of robust interfacial bonding with glass fibers, creating a continuous reinforcing network that facilitated efficient stress transfer under mechanical loading. This strengthening mechanism was further amplified at moderate GF loadings (10–20 wt%) through improved homogeneity of fiber dispersion and interfacial adhesion quality, which collectively enhanced damage resistance.

**Figure 9:**
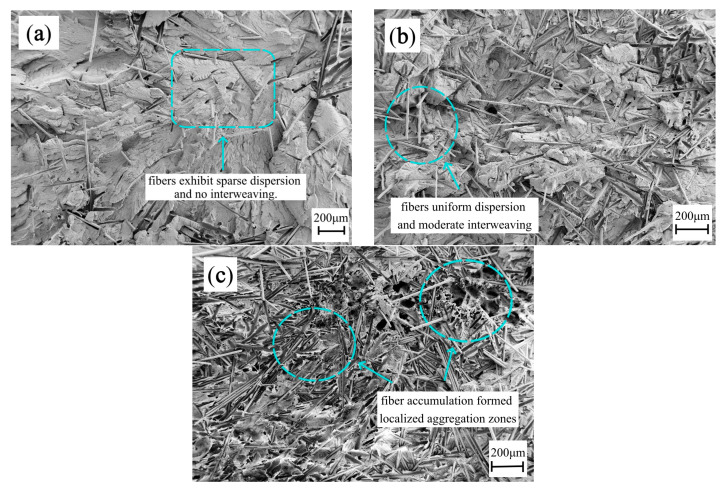
Fracture morphology of weld lines in PP-GF composites at different fiber contents: (**a**) 10 wt%; (**b**) 20 wt%; (**c**) 30 wt%.

Specimens with weld lines exhibited a distinct nonlinear mechanical response to increasing GF content. As shown in Figure 8, both their tensile and flexural strengths initially increased (for GF contents of 0–20 wt%) but declined sharply when the GF content exceeded 20 wt%; both tensile and flexural strengths initially increased (0–20 wt% GF) but sharply declined beyond 20 wt% GF. This nonlinearity is directly attributable to microstructural evolution within the weld line region (Figure 9a–c). At 20 wt% GF (Figure 9b), fibers exhibited uniform dispersion and moderate interweaving, facilitating effective stress transfer through robust fiber–matrix interfacial bonding. However, at 30 wt% GF (Figure 9c), excessive fiber accumulation near the weld line formed localized aggregation zones. These zones disrupted polymer chain entanglement by limiting melt penetration into the weld interface while simultaneously reducing interfacial contact area due to fiber clustering. The resultant stress concentration and premature interfacial debonding accounted for the observed strength degradation at high GF content.

The adverse effects of high GF content are further exacerbated by incomplete matrix wetting. At 30 wt% GF, the PP matrix failed to fully impregnate fiber bundles, resulting in voids and poor resin–fiber adhesion. This uneven distribution causes irregular stress concentrations, accelerating interfacial debonding. Additionally, high GF content restricts molecular diffusion and crosslinking at weld lines, preventing effective polymer chain entanglement. As molten streams converge, the discontinuity at the weld interface intensifies, reducing fracture resistance in high GF composites.

Weld line factors (*F_kl_*) quantify this degradation, as shown in Table 4. For PP-20GF composites, tensile *F_kl_* decreased from 0.99 (0 wt%) to 0.34 (30 wt%), while flexural *F_kl_* dropped from 0.96 to 0.38. Impact resistance suffers most severely, with *F_kl_* plummeting from 0.95 to 0.36, highlighting the disproportionate sensitivity of toughness to fiber accumulation. These trends underscore the dual role of GF content. Moderate levels (≤20 wt%) enhance bulk properties through fiber reinforcement, while excess content (>20 wt%) amplifies weld line defects through interfacial failure.

This behavior contrasts with findings in short-fiber composites, where Guo [24] reported linear strength gains with fiber content due to random fiber distribution mitigating alignment-induced weaknesses. In PP-GF, long fibers align parallel to weld lines under flow, creating continuous weak interfaces that dominate failure, which is a mechanism absent in short-fiber systems. Similarly, amorphous polymers, like PS/PMMA [24], achieve full weld line recovery via chain diffusion, a process suppressed in semi-crystalline PP-GF due to restricted chain mobility and crystallization-driven embrittlement.

The optimal GF content balances the reinforcement effect against weld line degradation. While 20 wt% GF maximizes bulk strength and interfacial bonding, higher concentrations exacerbate defects, particularly under impact loading. These findings emphasize the need for microstructure-driven design in PP-GF composites, prioritizing fiber dispersion and matrix–fiber adhesion to mitigate weld line risks in high-performance applications.

## 4. Simulation Results Analysis

### 4.1. Tensile Strength Prediction

**Figure 10:**
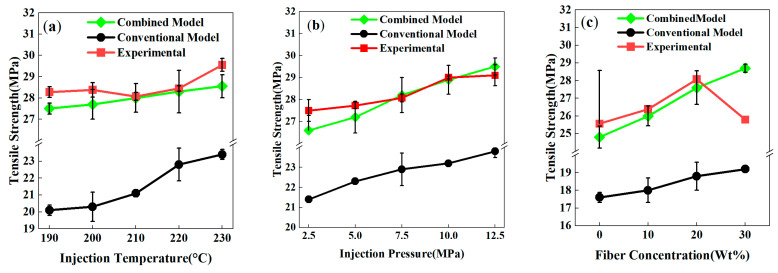
Joint simulation curves of the tensile strength of PP-GF composites under different process conditions: (**a**) injection temperature; (**b**) injection pressure; (**c**) fiber content.

Based on a Moldex3D–Abaqus coupled simulation platform, this study systematically investigated the regulatory effects of injection temperature, pressure, fiber content, and fiber length on the tensile strength of weld lines in PP-GF composites (Figure 10). The Hashin failure model, a widely used classic approach in composite mechanics, was chosen as the comparative benchmark (named as the conventional model). A comparison between the simulation results of the conventional failure model and the proposed combined model reveals that the combined model has significantly higher predictive accuracy than the conventional Hashin model across all process parameters. Its predictions are highly consistent with experimental results, verifying the effectiveness of the new model in characterizing the complex mechanical behavior of weld lines.

Specifically, Figure 10a shows that within the injection temperature range of 190 °C to 230 °C, the conventional model predicts a linear increase in tensile strength, whereas the combined model aligns with experimental observations. The simulated trend of tensile strength (slight increase) was consistent with the overall experimental trend (fluctuating around 28 MPa). Notably, the combined model successfully captures the non-monotonic characteristics of experimental data, yielding predictions closer to experimental values and significantly reducing the systematic underestimation caused by the conventional model.

As shown in Figure 10b, for the effect of injection pressure on tensile strength, the combined model’s simulations are highly consistent with experimental data across the entire pressure range (2.5–12.5 MPa). Particularly, in the medium-to-high pressure interval (7.5–12.5 MPa), the prediction error is minimal, confirming the model’s high accuracy in predicting melt compactness regulation by pressure parameters and the resulting mechanical property improvements.

Regarding the influence of material composition (Figure 10c), experimental validation indicates that when the fiber content is ≤20 wt%, the simulation results of the combined model are highly consistent with the experimental results in terms of both tensile strength trends and absolute values. However, a noticeable discrepancy arises at a GF content of 30 wt%. The combined model predicts a continued increase, whereas the experimental values decline. However, a noticeable discrepancy emerges at 30 wt% GF. The combined model predicts a continued increase, while experimental values decline. This discrepancy likely arises from the conflict between the simulation’s assumption of uniform fiber distribution and experimental observations of localized fiber aggregation in weld lines. Theoretical analysis suggests that fiber reinforcement in weld lines primarily depends on the fibers’ ability to span the interface; higher fiber content may exacerbate fiber orientation and aggregation while reducing the entanglement density of resin polymer chains—key mechanisms not fully characterized in the current simulation framework. In contrast, the conventional model, though capturing the same trend as the experiments, significantly underestimates the actual tensile strength.

The simulation discrepancy at high fiber content is also associated with altered fiber–fiber and fiber–matrix interactions. Increased fiber density intensifies fiber friction and entanglement and may trigger more interfacial debonding and fiber pull-out, thereby weakening reinforcement efficiency by restricting stress transfer paths. The experimental observation of decreasing tensile strength at 30 wt% GF (a non-monotonic response) is not fully replicated in existing simulations, partly due to the oversimplified treatment of complex interfacial mechanics and failure modes under high fiber content conditions.

This study demonstrates that the proposed joint simulation method provides reliable tensile strength predictions for PP-GF weld lines under moderate fiber content (≤20 wt%) and short-fiber conditions, with accuracy significantly superior to the conventional model. Future work should incorporate microstructural parameters such as fiber clustering degree, interfacial porosity, and fiber fracture probability and explore the establishment of a multiscale damage model for fiber–matrix debonding.

### 4.2. Stiffness Prediction

To evaluate the accuracy of the model in predicting the tensile stiffness within weld line regions, simulations were conducted for PP-GF composites under varying processing conditions (temperature, pressure, fiber content, and fiber length) based on a Moldex3D–Abaqus coupled simulation platform. The stiffness calculation methodology was rooted in micromechanics-based homogenization theory, which bridges the fiber–matrix microstructure to macroscopic elastic properties. Experimental and simulation comparisons demonstrate that the proposed multiscale combined model exhibits higher predictive accuracy for weld line stiffness than the conventional model.

Numerically, fiber orientation distribution (FOD) data obtained from Moldex3D simulations were used to generate three-dimensional representative volume elements (RVEs) with periodic boundary conditions in Digimat. The matrix (PP) and fiber (glass) were assigned isotropic (E_m_ = 1.5 GPa, ν_m_ = 0.35) and transversely isotropic (E_f_ = 72 GPa, ν_f_ = 0.22) material properties, respectively. Uniaxial tensile strain (ϵ_11_ = 1%) was then applied to the RVE in Abaqus, and the homogenized stress response was extracted to compute the macroscopic Young’s modulus E_11_.

**Figure 11:**
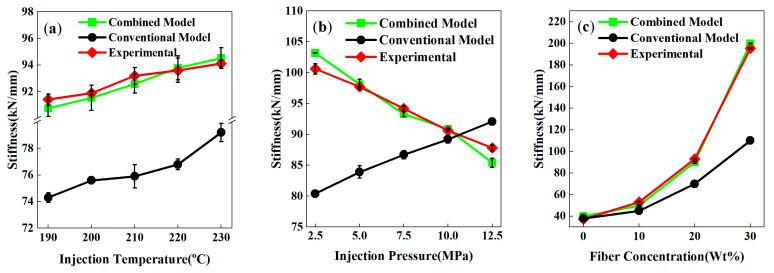
Joint simulation curves of stiffness of PP-GF composites under different process conditions: (**a**) injection temperature; (**b**) injection pressure; (**c**) fiber content.

The results revealed that as the injection temperature increased from 190 °C to 230 °C (Figure 11a), the stiffness values predicted by the combined model aligned with the experimental upward trend with small errors, while the conventional model significantly underestimated stiffness and failed to capture the temperature-induced performance enhancement. Regarding the effect of injection pressure (Figure 11b), the combined model successfully reproduced the experimental downward trend of stiffness as pressure rose from 2.5 MPa to 12.5 MPa, with good consistency in the 5–10 MPa range. In contrast, the conventional model predicted an opposite upward trend and yielded values notably lower than experimental data under medium-to-low pressure, indicating limitations in modeling the pressure–stiffness relationship.

Fiber content exerted the most significant influence on stiffness (Figure 11c). As fiber content increased from 0 wt% to 30 wt%, experimental stiffness improved substantially, and the combined model accurately captured this enhancement—its predicted trend was highly consistent with experiments, with a maximum error of less than 3.5%. While the conventional model could roughly track the trend for composites with low-to-medium fiber content, it significantly underestimated the strength at high fiber content (e.g., 30 wt%), indicating its inability to fully characterize the reinforcement mechanism under high fiber content conditions.

Furthermore, the combined model’s superiority over the conventional model was confirmed across diverse process parameters and fiber characteristics, with high reliability, particularly for moderate fiber content (≤20 wt%) and typical processing windows. This validates its potential for guiding complex molding processes involving weld lines while delivering reliable numerical data for real-world engineering applications. To further improve predictive accuracy for high fiber content systems, future work should incorporate fiber fracture models, more precise orientation distribution functions, and interfacial damage mechanisms.

### 4.3. Flexural Strength Prediction

Simulations of flexural strength in PP-GF weld line composites revealed a lower agreement with experimental data compared to tensile strength predictions—both the combined model and conventional model yielded significantly underpredicted values, indicating substantial room for improvement in existing predictive frameworks. This discrepancy stems from limitations in current models and the inherent complexity of flexural behavior, which involves coupled tensile, compressive, and shear responses, as well as intricate interactions between fiber orientation, interfacial strength, and matrix toughness.

**Figure 12:**
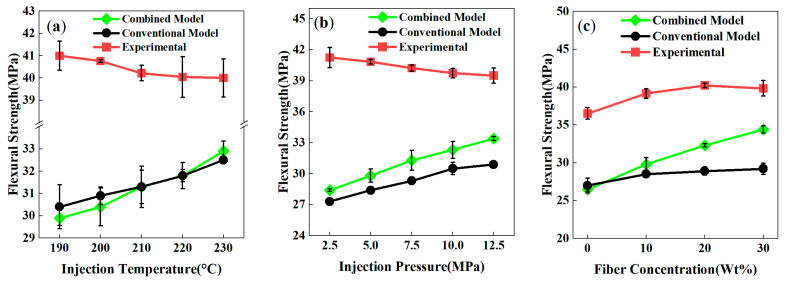
Joint simulation curves of flexural strength of PP-GF composites with weld lines under different process conditions: (**a**) injection temperature; (**b**) injection pressure; (**c**) fiber content.

As shown in Figure 12a, when the injection temperature increased from 190 °C to 230 °C, predictions from both the combined model and conventional model exhibited a slight upward trend with an increase of no more than 3 MPa; in contrast, experimental values remained stable around 40 MPa with a slight decline. The absolute deviation between model predictions and experimental data exceeded 8 MPa, reflecting insufficient capture of the coupled mechanisms of temperature-induced crystallization evolution and interfacial healing in current simulations.

The influence of injection pressure on flexural strength is illustrated in Figure 12b. As pressure increased from 2.5 MPa to 12.5 MPa, the combined model and conventional model predicted approximate linear increases of ~5 MPa and 3.6 MPa, respectively. Conversely, experimental values decreased gradually from 41.25 MPa to 39.5 MPa, demonstrating that high pressure may induce polymer chain degradation or fiber fracture due to intensified melt shear, physical effects not yet integrated into existing models.

The effect of fiber content further highlighted model limitations (Figure 12c). Although the combined model’s predictions were closest to the experimental results at 20 wt% fiber content, neither model reproduced the experimental non-monotonic trend. Flexural strength slightly declined at 30 wt% GF, likely due to stress concentration and interfacial weakening caused by fiber aggregation. The conventional model consistently underestimated flexural strength with a flat response across the entire fiber content range, demonstrating its inability to effectively characterize the flexural behavior of fiber-reinforced composites.

The lower prediction accuracy for flexural strength arises from the interplay of unmodeled phenomena rooted in the material’s multiaxial stress state under flexural loading. Unlike tensile strength, which benefits from dominant fiber alignment along the loading direction, flexural performance is governed by complex interactions between off-axis fibers (perpendicular to the neutral axis), matrix ductility, and interfacial debonding resistance. Current models—including the combined model and conventional model—remain based on uniaxial stress assumptions or simplified fiber orientation tensors, failing to fully characterize fiber redirection, progressive interfacial debonding, and local plastic deformation during actual flexural processes. This shortcoming is exacerbated under high fiber content and long-fiber conditions, where fiber–matrix interactions, fiber fracture, and non-uniform orientation distribution further increase modeling complexity.

**Figure 13:**
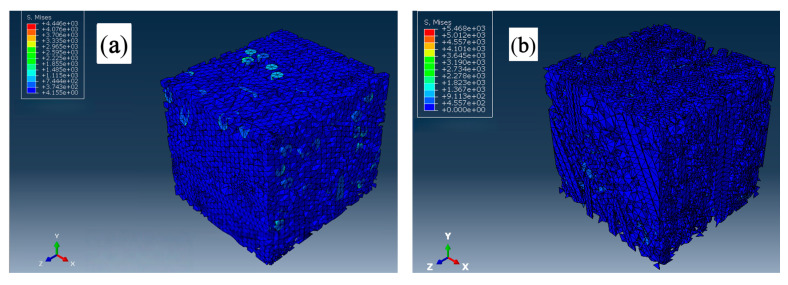
Mises stress distribution in the PP-GF composite RVE: (**a**) under tensile stress; (**b**) under flexural stress.

The Mises stress distribution intuitively reflected the differences in stress distribution of the PP-GF composite material RVE under different load conditions, as illustrated in Figure 13. Under the load of tensile stress (Figure 13a), the PP matrix first reached the yield strength. As the load gradually increased, the failed matrix elements were deleted, while the glass fiber maintained structural integrity and load-bearing capacity. This phenomenon reflected the typical mechanical mechanism of “fiber dominated load-bearing” of composite materials. Thus, the present model displayed a high simulation accuracy under tensile loads. However, under the load of flexural stress (Figure 13b), the Mises stress difference between fibers and the matrix is significantly smaller than that under tensile stress conditions, indicating that the fibers displayed a lower load-bearing capacity. This is mainly caused by the ignorance of the shear stress of fibers in the present model. Thus, the underestimated load-bearing capacity of fibers under flexural load finally resulted in lower simulated flexural strength.

To enhance model accuracy, future work must focus on introducing multiscale modeling strategies, including the integration of microstructurally realistic fiber distribution, interfacial damage evolution laws, and strain rate-dependent constitutive models. A refined framework should explicitly resolve fiber–matrix interactions in shear and compression while coupling molecular-scale chain dynamics with mesoscale fiber network mechanics to address gaps in current simulations.

## 5. Conclusions

This study investigated the mechanical properties of weld lines in injection-molded glass fiber (GF)-reinforced composites and built a mathematical model to predict these properties. The influences of injection temperature, injection pressure, and fiber content on the mechanical properties and failure mechanism of weld lines were investigated. Combining polymer chain dynamics with fiber–matrix interfacial debonding mechanics, a mathematical model was developed to predict the weld line strength. Multiple simulation software and tools were integrated in the analysis, including Moldex3D for mold flow analysis, Digimat for material modeling, and Abaqus for multiscale mechanical analysis. The simulation and experimental results were further compared and analyzed to validate the accuracy of the model. The following main conclusions can be drawn.

(1) Injection temperature, injection pressure, and fiber content all affect the mechanical properties of weld lines, with fiber content exerting the most significant influence. When the fiber content exceeds 20wt%, the tensile and flexural strengths of weld lines decrease sharply compared with specimens without weld lines. In the weld line region, the fibers align parallel to the fracture surface and form aggregates, providing a very weak reinforcing effect to the matrix.
(2) A novel multiscale simulation framework was developed by integrating polymer chain diffusion dynamics (Flory–Huggins free energy theory) and orientation-dependent fiber–matrix interfacial debonding mechanics, which, for the first time, realizes the coupling of semi-crystalline polymer dynamics and long-fiber anisotropy and solves the defects of traditional models that ignore intermolecular chain interactions and crystallization kinetics–fiber orientation coupling. The framework integrates Moldex3D-derived fiber orientation data, Digimat microstructure reconstruction, and Abaqus multiscale analysis and clarifies the influence of the laws of injection temperature, pressure, and fiber content on the mechanical properties of weld lines.
(3) Experimental validation shows that the model has high prediction accuracy for the tensile strength and stiffness of weld lines in PP-GF composites with an error of less than 10% and 3.5%, respectively. The industrial optimization directions derived from the research include increasing the melt temperature to enhance chain diffusion, moderating the injection pressure to suppress excessive fiber alignment, and controlling the fiber content within 20wt% to balance the reinforcement effect and weld line defects. This study provides effective simulation tools for the weld line control and performance optimization of fiber-reinforced composites.

## Figures and Tables

**Table 1:** Nominal material properties of PP and PP-30GF.

Properties	Test Standards	Value		Unit
		PP	PP-30GF	
Density	ASTM D792	0.91	1.125	g/cm^3^
Tensile strength	ASTM D638	32	61.4	MPa
Flexural strength	ASTM D790	36	120	MPa
Young’s modulus	ASTM D638	1500	6500	MPa

**Table 2:** Weld line factors of PP-20GF composites at different injection temperatures.

Injection Temperature	*F_kl_* of Tensile Strength	*F_kl_* of Flexural Strength
190 °C	0.55 ± 0.03	0.52 ± 0.05
200 °C	0.54 ± 0.01	0.53 ± 0.02
210 °C	0.54 ± 0.02	0.54 ± 0.03
220 °C	0.53 ± 0.03	0.60 ± 0.01
230 °C	0.55 ± 0.04	0.61 ± 0.01

**Table 3:** Weld line factors of PP-20GF composites under different injection pressures.

Injection Pressure	Fkl of Tensile Strength	Fkl of Flexural Strength
2.5 MPa	0.52 ± 0.02	0.57 ± 0.04
5 MPa	0.52 ± 0.01	0.56 ± 0.02
7.5 MPa	0.54 ± 0.01	0.54 ± 0.03
10 MPa	0.55 ± 0.05	0.53 ± 0.02
12.5 MPa	0.55 ± 0.02	0.52 ± 0.01

**Table 4:** Weld line factors of PP-GF composites at different fiber contents.

Fiber Contents	*F_kl_* of Tensile Strength	*F_kl_* of Flexural Strength
0 wt%	0.99 ± 0.06	0.96 ± 0.01
10 wt%	0.74 ± 0.03	0.75 ± 0.01
20 wt%	0.53 ± 0.02	0.54 ± 0.03
30 wt%	0.34 ± 0.02	0.38 ± 0.02

## Data Availability

The original contributions presented in this study are included in the article. Further inquiries can be directed to the corresponding authors.
